# Did a small thermosensitive intron contribute to the temperate adaptation of *Drosophila melanogaster*?

**DOI:** 10.18103/mra.v11i11.4624

**Published:** 2023-11

**Authors:** Isaac Edery

**Affiliations:** Center for Advanced Biotechnology and Medicine, Department of Molecular Biology and Biochemistry Rutgers University

## Abstract

*Drosophila melanogaster* was first used for research in the early 1900’s by scientists located in the northeastern corridor of the United States, gaining prominence with the establishment of the famous “fly room” by Thomas Hunt Morgan at Columbia University circa1908. Several reasons for using *D. melanogaster* in research are well known; easy and inexpensive to breed, short lifespan, amongst others. But why was this insect species flourishing in a temperate northeast region of the New World during the late 1800’s when they originated in the tropical forests of sub-Saharan Africa millions of years ago? The purpose of this review is to provide an overview of the experimental underpinnings for a temperature sensitive mechanism that likely contributed to the rather unique ability of *Drosophila melanogaster* to successfully colonize temperate regions on a global scale. It also furnishes an interesting historical insight into how ancestral genetics serendipitously held the keys to the journey of *D*. *melanogaster* becoming such a popular research organism. While numerous papers have been published detailing different aspects of the work, this is the first comprehensive review. Herein, I discuss the discovery of a small thermosensitive intron in *D. melanogaster* (termed dmpi8) that controls midday siesta levels. Like many day-active animals, *Drosophila* exhibits a robust genetically based midday siesta that is protective in warm climates. Yet long bouts of daytime inactivity might be counterproductive in temperate climates, especially since daylength in these regions is shorter during the cooler months. Evidence discussed in this review strongly indicates that targeting of dmpi8 splicing efficiency by natural selection enhanced the ability of *D. melanogaster* to scale daytime sleep levels commensurate with a wide range of local climates. Surprisingly, dmpi8 splicing regulates midday siesta levels in *trans* by controlling the expression of a nearby anti-siesta gene called *daywake*. The “fortuitous” genetic arrangement of a thermosensitive intron in proximity to an anti-siesta gene might have contributed to the cosmopolitan nature of *D. melanogaster* and its historical journey in becoming a popular research organism.

## Introduction

The genus *Drosophila* contains around 1,600 described species that vary in many attributes and are found all over the world from tropical and desert, to temperate and alpine regions^1^. Of all these species, *D. melanogaster* has a unique position in the scientific literature, a *prima-donna* subject of human inquiry. A recent search of Pubmed using *“Drosophila melanogaster”* as the query returns over 60,000 citations, whereas the next highest, *Drosophila simulans* barely cracks 1,500. Five Nobel Prizes have been awarded to nine scientists for work primarily done using *Drosophila melanogaster* as a model organism. Studies using *D. melanogaster*, have been foundational in understanding evolution, genetics, development and neurobiology (e.g.,^2,3^). By combining cutting-edge computing and imaging, it is now possible to visualize 3-D interactive maps of every single neuron and synapse in the adult brain of *D. melanogaster*. How did *D. melanogaster* come to be such a favorite organism for probing minds, from high school students to the world’s most prestigious research institutes?

The answer to this question begins in the United States towards the start of the 20^th^ century. Serendipity, exchange of ideas, re-discovery of Mendelian genetics, interest in a nagging question and constraints imposed by the size of a room all played major parts. Charles Woodworth, an entomologist, is the first scientist credited with breeding *Drosophila melanogaster*, introducing it to William Castle at Harvard whose work inspired Thomas Morgan, then at Columbia University (for historical reviews, see^3–6^). This series of events led to the first “Fly Room”. Morgan was attracted to the easy and cheap breeding of *D. melanogaster* that could be maintained in large numbers with the help of students even in a small room. Fast breeding times, large numbers and easily recognized physical phenotypes were ideal for experimental probing into the basis of heredity. Morgan and colleagues isolated a white-eyed mutant in 1910, following that up with the discovery of many more mutants and established chromosomes as key units of inheritance—for which he won a Nobel Prize in 1933. Perhaps appropriately, the *white* mutatant is the most widely used laboratory strain for *Drosophilist*, serving as host for generating tens of thousands of genetically altered strains by simply following the co-rescue of the wildtype red eye color. Indeed, with the demonstration that transgenic *D. melanogaster* could be generated by P-element transformation in the early 1980’s^7,8^, this solidified its place as a workhorse animal model system, which continues unabated with the development of ever-more innovative genetic tool kits and realms of data to be mined.

Yet a much different story might have occurred if *D. melanogaster* was not already endemic to parts of the United States where these early pioneers set up shop. Sure, nowadays wherever you might be, these flies have likely pestered your kitchens and buffet tables, especially those with ripening fruit. But that was not always the case, at least not in the United States. *D. melanogaster* was first described by the German entomologist Johann Wilhem Meigen in 1830. The first report of *D. melanogaster* in the United States was in the northeast in 1875, quickly radiating west over the next few decades^4^. Irrespective of when *D. melanogaster* first arrived in the New World, it was already a cosmopolitan strain that closely associated with humans.

**Fig 1.**
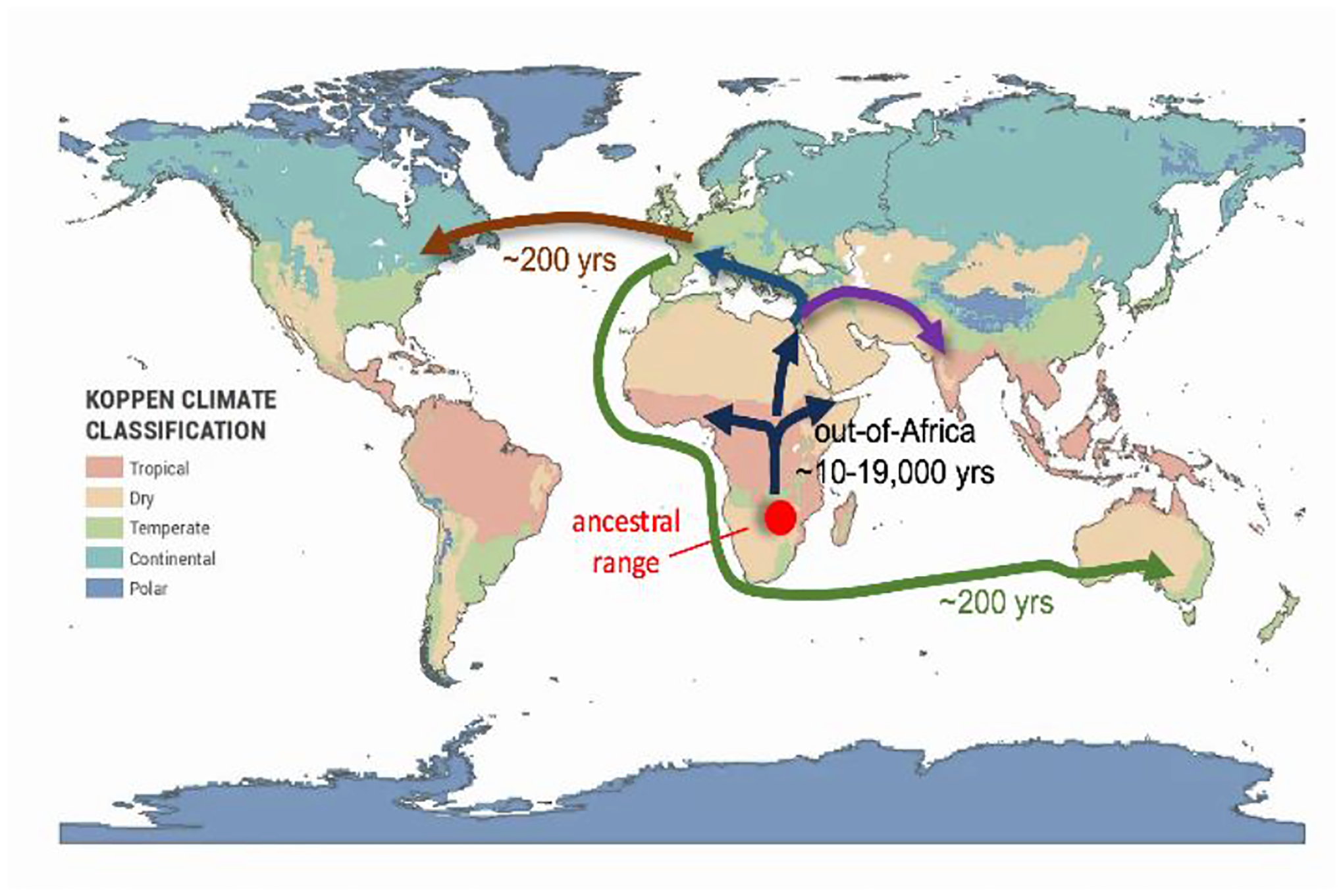
Proposed historical expansion of *D. melanogaster* beyond its ancestral range in Africa (red circle), with emphasis on the more recent colonization of North America and Australia. Map based on EarthHow.com; *Drosophila melanogaster* migration routes based on refns.^13,15^

It is thought that the genus *Drosophila* originated in the lowlands of sub-Sahara tropical Africa approximately 40–50 million years ago^9–11^ (Fig. 1). More recent findings have fine-tuned this hypothesis and suggest *D. melanogaster* originated in the tropical forests of current day Southwestern Africa (Zimbabwe and Zambia)^12,13^. Expansion of *D. melanogaster* into Europe and Asia is thought to have occurred via a single “out-of-Africa” bottleneck some 12–19,000 years ago, around the time of the last ice age, followed by a quick population expansion throughout Africa and Eurasia^14,15^. The America’s, Australia, Japan and other places separated by large bodies of water that experienced more recent human migration or commerce from Africa and Eurasia, have several 100 years at most in providing a home for naturally-breeding populations of *D. melanogaster*. This tropical species is now endemic to temperate regions and high altitudes around the world.

Given the diversity of *Drosophila* it is not surprising that these species adapted to survival in geographical locations that differ greatly in temperature, humidity, diets, etc. However, even for the closely related nine species in the *D. melanogaster* species subgroup (*erecta, mauritiana, melanogaster, orena, santomea, sechellia, simulans, teissieri* and *yakuba*), only *D. melanogaster* and *D. simulans* are cosmopolitan and apparently opportunistic in human commensalism^16^. Of the two, *D. melanogaster* is easily more widespread around the globe. How and when *D. melanogaster* became a human commensal is not clear.

It’s presumed ancestral range around Zimbabwe (Fig. 1) is associated with the marula fruit, a resource that even today’s cosmopolitan strains prefer^12^. It is proposed that the harvesting of large quantities of marula fruit by indigenous peoples of Southwestern Africa some ~10,000 years ago might explain the commensalism. Intriguingly, large supplies of marula fruit were kept in caves, which may have seeded the current ability of *D. melanogaster* to share indoor living with humans.

While adapting to cohabitating with humans and becoming a generalist or opportunistic feeder certainly helps explains its worldwide distribution, there is more to the story. Most notably, natural populations of *D. melanogaster* show extensive genetic differentiation as a function of latitudinal and altitudinal clines that show parallel changes, patterns consistent with temperature as the driving selection pressure (reviewed in,^17–19^). Additionally, there is genome-wide correlation between clinal and seasonal variation in natural *D. melanogaster* populations suggesting common and dynamic mechanisms in establishing fluctuations in allele frequencies that are selected for real-time climate adaptation^20^. Indeed, the direction of allele frequency change at seasonally variable polymorphisms can be predicted by weather conditions in the weeks prior to sampling^21^.

These studies not only indicate that adaptation to local temperatures increases the fitness of even cosmopolitan *D. melanogaster* strains, but that it can occur rapidly, suggesting a rich source of historically sustained genomic variants within these populations that can be readily selected in distinct combinations to optimize organismal function to local temperature ranges. In this regard it is important to note that the presumed ancestral range of *D. melanogaster*, while tropical, sits close to the edge of the *Tropic of Capricorn*. Seasonal temperatures can fluctuate between lows of 9–18° to highs of 24–30°C depending on altitude. Thus, it is likely that even in its ancestral range, *D. melanogaster* as an ectotherm had to find flexible strategies to adapt to long periods of cooler and warmer temperatures. Therefore, although the world-wide expansion of *D. melanogaster* to temperate regions beyond its ancestral range is due to its association with humans, adapting to local temperatures plays a key role in enhancing the fitness of natural populations. One of those traits, and the subject of this review, is sleep. Specifically, midday sleep, or more commonly referred to as “siesta” (meaning sixth hour or midday).

### Temperature, daily activity patterns and midday siesta.

Virtually all animals exhibit some form of sleep or quiescent state that has a characteristic timing during a daily cycle and length. In general, invertebrate and vertebrate animals are mainly active at particular times of day, most notably during daylight hours (diurnal), the night (nocturnal), dawn/dusk (crepuscular) or cathemeral (both day and night)^22,23^. Patterns of daily activity are mainly constrained by duration of illumination and thermal limits suitable for organismic function. Daily wake-sleep cycles and their timing are governed by internal cell-based circadian (~24 hr) clocks^24–27^. Sleep is also regulated by homeostatic pathways, where sleep drive builds with length of wake^28–30^. In addition, environmental cues, biotic factors and internal physiological state (e.g., hunger) have major influences on wake-sleep behavior (e.g., ^31^).

Ambient temperature features prominently in sleep behavior (^32–35^). Ever try to sleep at night when it’s hot? Early studies analyzing garter snakes showed that ambient temperature has strong effects on the daily activity patterns of animals^36,37^. On cool days snakes exhibit peak activity during the middle of the day which is suppressed at higher temperatures where they are mainly active in late night-early morning. Many day-active animals whether they can actively thermoregulate their body temperature (homeotherms) or not (poikilotherms) avoid excess activity during the middle of hot days. The increase in midday quiescence on hot days is commonly referred to as siesta. Although largely abandoned in the modern world, studies of several preindustrial societies showed that midafternoon napping increased in frequency and duration during the summer compared to winter^38^. Like humans, nighttime sleep in *D. melanogaster* is more fragmented and less intense at warmer temperatures, whereas daytime sleep intensity increases with temperature^39–42^ (further discussed below).

Midday siesta aligns well with the timing of post-lunch drowsiness or postprandial sleep/fatigue, which is widely observed irrespective of temperature^43–45^. This early afternoon drowsiness is essentially a feature of the dynamics underlying the circadian timing system and sleep homeostatic pathways^46,47^. Depending on the time-of-day, the balance between sleep drive and wake drive fluctuates. Your circadian clock strongly pushes wake from post afternoon to early nighttime hours. Thus, midday is a more vulnerable state for sleepiness, which can be enhanced by heat, feeding and possibly other factors. While midday siesta is most associated with avoiding heat, there are other environmental hazards, most notably exposure to noxious levels of radiation from UV and/or blue light. Moreover, the dangers of heat/UV exposure are generally more severe for smaller animals since they have a larger surface-to-volume ratio making them at increased risk for water-loss and desiccation.

Many studies have shown the benefits of short daytime napping (“power-napping”), such as improving cognitive functions and lowering blood pressure^48^. However, excessive daytime napping or sickness behavior is linked to poor prognosis for many medical disorders, such as Parkinson’s, Alzheimer’s and diabetes^49,50^. Intriguingly, daytime skin temperature for individuals with several of these disorders are higher than usual, reinforcing the notion that elevated daytime body temperature is sleep promoting^51,52^. Large scale analysis of genetic variation in human sleep behavior reveals that nighttime and daytime sleep are governed by different and/or non-overlapping pathways, serving different functions^53–55^. Indeed, studies in *Drosophila* have clearly established different mechanisms that underlie daytime and nighttime sleep^56^.

### Discovery of the thermosensitive dmpi8 intron regulating midday siesta in *D. melanogaster*.

Some 25 years ago we sought to understand how seasonal changes in temperature affect the distribution of daily activity patterns in *D. melanogaster*, studies inspired by earlier work analyzing garter snakes (see above). We hoped to use the genetic tools available in *Drosophila* to gain mechanistic insights; essentially, are the changes in activity patterns an acute reaction to ambient temperature or involve an underlying thermosensing mechanism?

The standard laboratory conditions for analyzing daily wake-sleep behavior in *Drosophila* is based on measuring fly locomotor activity levels. The standard lab conditions are maintaining flies for several days at 25°C while exposed to 12hr light: 12hr dark cycles [LD; where Zeitgeber time 0 (ZTO) is defined as lights-on]^57^. Subjecting *Drosophila* to days of 12 hr light followed by 12 hr dark is reasonable considering they originated around equatorial Africa. In the classic experimental design, individual flies are placed in small glass tubes whereby locomotor activity movement can be tracked by counting “beam brakes” using an infra-red source positioned across a photomultiplier tube. This system was refined and marketed by a small company (at that time) called Trikinetics that was situated a short distance from the labs of Drs. Hall and Rosbash at Brandies University in Waltham, Massachusetts, USA. Working together in the early 1980’s, they optimized the “Drosophila Activity Monitor” (or DAM) system. Since then, other systems have been developed^58,59^ but the Trikinetics DAM system is still the most popular due to its ease of use and scalability. Indeed, numerous central clock genes were identified using the Trikinetics system in large-scale mutant screens, playing an assisting role in the 2017 Nobel Prize for Medicine and Physiology awarded to Drs. Hall, Rosbash and Young (Rockefeller University) for ground-breaking discoveries on the mechanisms underlying circadian rhythms^60^.

**Fig. 2.**
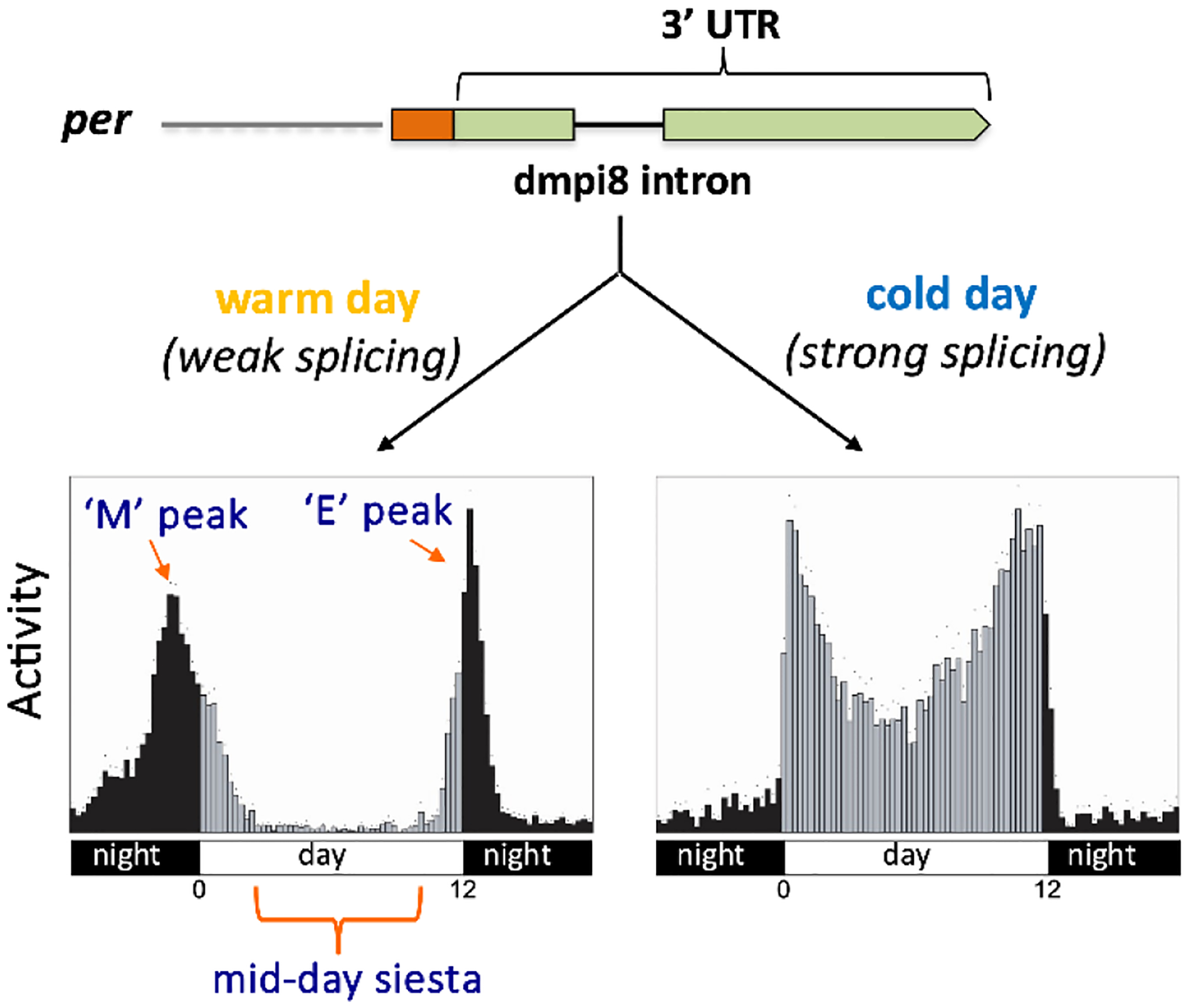
Thermal adaptation of midday siesta levels in *D. melanogaster* is regulated by thermosensitive splicing of the dmpi8 intron found in the *per* 3’ UTR. M = morning peak of activity; E = evening peak of activity.

We found that the daily distribution of activity in *D. melanogaster* is heavily influenced by ambient temperature^41^ (Fig. 2). At the standard temperature of 25°C, *D. melanogaster* exhibit two prominent clock-controlled activity peaks, a “morning” peak (M) centered around the lights-on transition and an “evening” peak (E) centered around the light-off transition^61^ (Fig. 2, bottom) At cooler temperatures, flies show increased daytime activity whereby the morning and evening peaks are closer together with elevated activity levels during the midday^41^. As temperatures increase, the morning activity peak shifts into the late night, whereas the evening peak is delayed into the early night with a concomitant large dip in midday activity^41,62^.

Since clock mechanisms not only control the pace of daily rhythms but also its phasing, we initially assumed temperature-induced changes in the oscillatory dynamics of central clock proteins was the basis for the thermosensitivity in daily activity patterns. A key mechanistic logic of circadian clocks is that some attribute in the expression of one or more core clock genes (e.g., levels, phosphorylated state) oscillates with an approximately 24 hr period that is inextricably linked to clock progression and rhythm generation^63,64^. Thus, we focused on circadian genes, especially *period* (*per*), critical to setting the pace and phase of the clock^65^.

The most relevant observation we made was that the splicing efficiency of a small intron (86–89 bp, depending on fly strain) in the 3’ untranslated region of *per* was modulated by temperature, being more efficient at colder temperatures^41^ (Fig. 2, top). We eventually called this intron dmpi8 (*D**rosophila* *m**elanogaster* *p**er* intron 8). Transgenic flies where the small 3’ UTR intron was either missing or could not be spliced both led to a similar delay in evening activity and longer midday siesta^41^. This suggests that *active* splicing *per se* and not just the presence or absence of the dmpi8 intron is the key molecular signal modulating daily activity patterns. Based on our findings that splicing of dmpi8 advanced the phase of *per* mRNA and protein levels^41^ we proposed a “clock-centric” explanation for the function of this intron in the thermal adaptation of daily activity patterns. Essentially, we postulated that the cold-enhanced splicing of dmpi8 advances daily cycles in *per* mRNA and protein levels leading to an earlier ‘evening’ activity which therefore decreases the duration of midday dip in activity^41^. As reviewed below, our initial *per*-based model turned out to be wrong because we had not anticipated the discovery of nearby gene that was indirectly regulated by dmpi8 splicing.

### Multiple weak splicing signals and thermosensitivity.

Comparative biology has been instrumental in the study of adaptation. Using this approach, we asked if thermal plasticity in siesta is also present in closely related *Drosophila* species that are only indigenous to their ancestral range in tropical Africa. As an initial test case we examined *D. yakuba*, which diverged from *D. melanogaster* about 5 million years ago^66^. Unlike *D. melanogaster*, midday siesta in *D. yakuba* is always prominent even at cool temperatures^67^. Intriguingly, a small intron is also present in the 3’ UTR of the *per* gene in *D. yakuba* (termed dyp3’), however its splicing efficiency is not modulated by temperature, consistent with the lack of thermo-responsiveness in siesta. This thermal difference in splicing efficiency between dmpi8 and dyp3’ introns was recapitulated in a simplified tissue culture system indicating that all the signals required for thermosensitive splicing are contained within the intron and nearby flanking regions^67^.

**Fig. 3.**
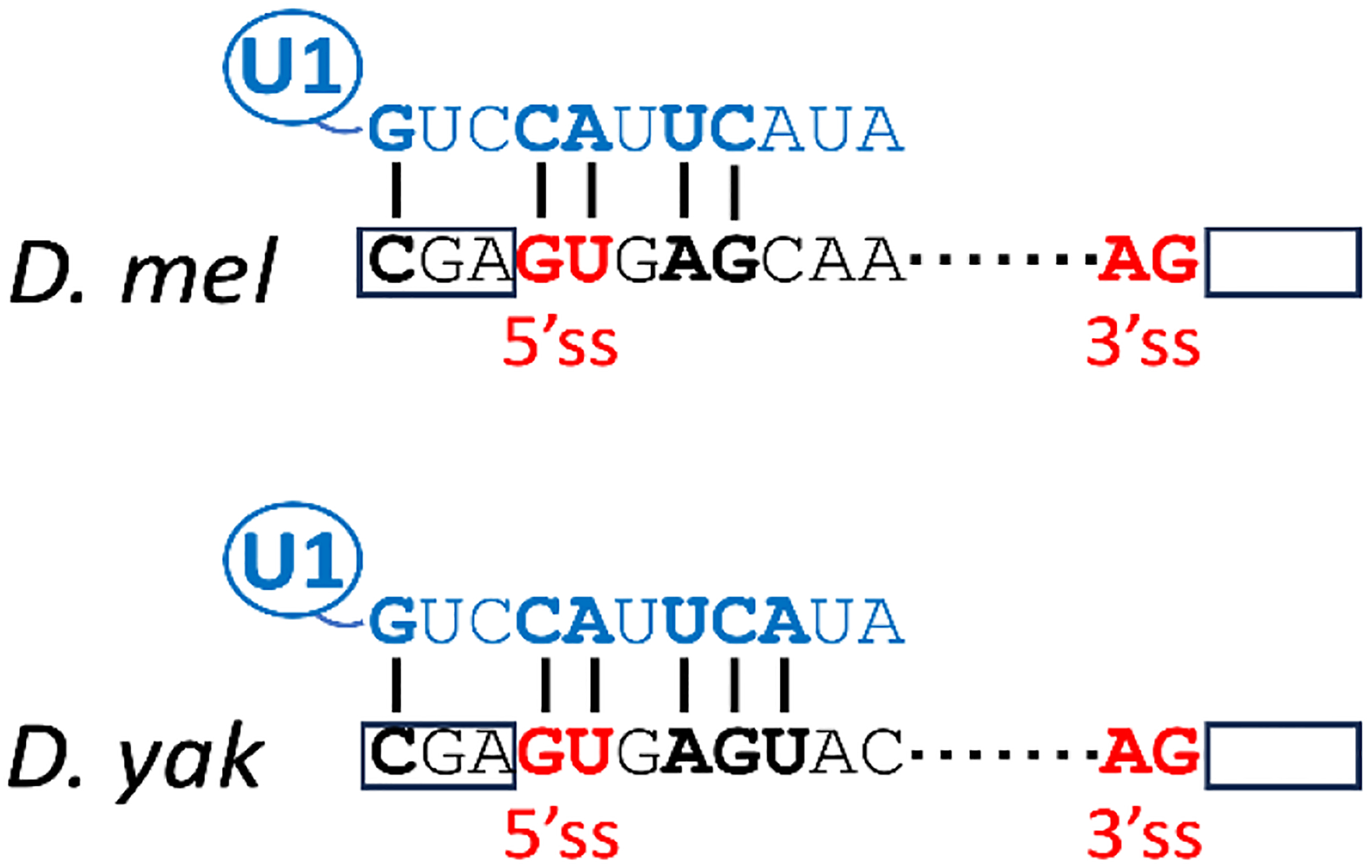
Weak 5’ss based on only 5 bp interactions with U1 contributes to thermal sensitivity in dmpi8 splicing efficiency, whereas the 6 bp for the *D. yakuba* intron is not thermosensitive for U1 binding within physiological temperatures.

Comparison of dmpi8 and dyp3’ showed that the 5’ and 3’ splicing signals for dmpi8 are extremely weak^67^ (Fig. 3). The first and rate-limiting step in pre-mRNA splicing is the recognition of the 5’ss by U1 snRNA and associated protein factors, which generally involves 5–8 base-pair interactions between the −3 and +6 nucleotides of the 5’ss^68–70^. The consensus 5’ss in *D. melanogaster* is (−3)MAGGTAAGT(+6) (where M=any nucleotide; GT= 5’splice site, where G=position +1)^71,72^. Optimizing the 5’ss of dmpi8, especially in conjunction with a stronger 3’ splice site, increased dmpi8 splicing efficiency and abolished its thermal sensitivity^67^. Importantly, transgenic flies wherein dimpi8 was replaced by the same intron with optimized 5 and 3’ splice sites (termed dmpi8UP) manifest reduced siesta compared to wildtype controls (termed dmpi8WT) at all temperatures tested. Intriguingly, the dmpi8 5’ss has the potential for 5 bp interactions with U1 snRNA, whereas the *per* 3’ intron from *D. yakuba* has an extra base pairing at the critical +6 position^67^ (Fig. 3). Presumably, the 5 base-pairing interactions between U1 snRNA and dmpi8 is sufficiently destabilized at higher but physiologically relevant temperatures to decrease spliceosome binding and overall splicing rate below that occurring at cooler temperatures. In contrast, the 6 base-pairing interactions between U1 snRNA and dyp3’ are not rate-limiting within the physiologically relevant temperature range for this species. Thus, it is possible that the absence of one critical base-pairing interaction between the 5’ss of dmpi8 and U1 snRNA facilitated the world-wide expansion of *D. melanogaster* to temperate regions by providing a thermally sensitive molecular throttle operating within physiologically relevant temperature ranges.

*D santomea* and *D. simulans* flies were also analyzed in the same study^67^. *D. santomea* diverged from *D. yakuba* about 400,000 years ago and is endemic to São Tomé, one of the Gulf of Guinea islands in west-equatorial Africa^73^. Whereas *D. santomea* flies exhibit little to no changes in siesta as a function of temperature, *D. simulans* does. Consistent with our model, the *per 3*’ intron in *D. santomea* is flanked by strong splice sites and shows constant high splicing efficiency over a range of temperatures, in sharp contrast to the *per*3’ intron in *D. simulans*. Thus, at least for the *D. melanogaster* subgroup, there is a strong correlation between multiple weak splice sites flanking a small intron in the 3’ UTR of *per*, thermosensitive splicing and adaptability of siesta levels to changes in ambient temperature. In temperate climates, cooler seasons are associated with shorter days. Therefore, the cold-enhanced splicing efficiency of dmpi8 might allow *D. melanogaster* to fulfill its daytime activities despite short days, giving it an advantage over flies that are hard-wired to sleep during the midday despite favorable thermal conditions^67^.

Importantly, just because *D. yakuba* and *D. santomea* appear limited in decreasing midday siesta on cool days, they do show thermal adaptation in other behaviors. For example, *D. yakuba* prefers warmer temperatures (mean = 25.47 °C) compared to *D. santomea* (22.56 °C), consistent with their distribution to either low altitudes or higher elevation, respectively^74,75^. Indeed, this climate-based spatial segregation may have contributed to the reproductive isolation and speciation of *D. yakuba* and *D. samtomea*^75^. Studies in *D. melanogaster* have uncovered the molecular basis for thermal preference, which is based on temperature sensitive neural circuits^39,76^, a mechanism distinct from how dmpi8 splicing modulates midday siesta. Clearly, different mechanisms underlie a multitude of behavioral adaptations to temperature that differ in selective pressures depending on variant life history traits even in closely related species. Thus, a species like *D. santomea* shows evidence of adaptation to cooler temperatures but this apparently did not extend to strong plasticity in midday siesta. Might it be that for closely related species to *D. melanogaster*, the ‘only’ adaptive route to reducing midday siesta on cold days is dependent on having a small intron in the 3’-terminal of *per* with multiple weak splice sites as a basis for thermosensitive splicing efficiency?

Although not a focus of this review, that multiple weak splicing signals might underlie thermosensitive splicing was strongly supported by earlier work from Murphy and co-workers using the Moloney murine sarcoma virus ts110 mutant that shows temperature sensitivity in growth^77–79^. It was suggested that weakened RNA:RNA interactions between key splicing signals and snRNPs might diminish spliceosome binding at higher temperatures. An estimated ~15% of all disease-associated mutations in humans affect splice sites^80–83^, with positions −1 and +5 likely to be most critical to U1 binding^68,84^. Disease-causing mutations that weaken the 5’ss can lead to temperature sensitive splicing, as shown for an allele of Ehlers-Danlos syndrome Type VII^85^. In comparing the 5’ss of the *per* 3’-terminal intron from *D. melanogaster* and *D. yakuba*, the former is missing a base-pairing interaction at +6 with U1 snRNA. Mutations at intronic position +6 or position +3 of U1 snRNA (which base-pairs with the intronic +6 site) lead to weakened splice sites that are casually linked to several human diseases, such as familial dysautonomia^86^, Ehlers-Danlos syndrome^87^ and medulloblastoma^88^. Perhaps many of the splicing mutations that weaken strong 5’ss have thermal sensitive phenotypes since these sites might lack other compensatory elements to ensure robust recognition. Intriguingly, the interaction between microRNAs and mRNAs are also based on a similar number of base-pair interactions as that of U1 snRNA and the 5’ss^89^. This suggests that the RNA:RNA interactions key to pre-mRNA splicing and miRNA regulation are ideally suited for thermal responsiveness to temperature ranges widely observed on Earth, either on a daily or seasonal scale.

### Single nucleotide polymorphisms, dmpi8 splicing and midday siesta.

As discussed above, midday siesta makes biological sense on hot days. However, on cool days sleeping during the middle of the day for a visual day-active organism might prove counter-productive. We reasoned that *D. melanogaster* adapted to cooler regions would exhibit smaller mid-day siestas compared to their warm adapted counterparts. *Drosophila* is one of the best studied species in clinal studies. These flies have been captured by many different researchers over the years and usually kept in private laboratories. The different developmental, phenotypic and behavioral traits measured as a function of geographical location are stable despite years of laboratory rearing^19,90^. In general, latitudinal and altitudinal clines are ascribed to differences in temperature (although other factors, such as oxygen tension is also relevant, especially for altitude).

**Fig. 4.**
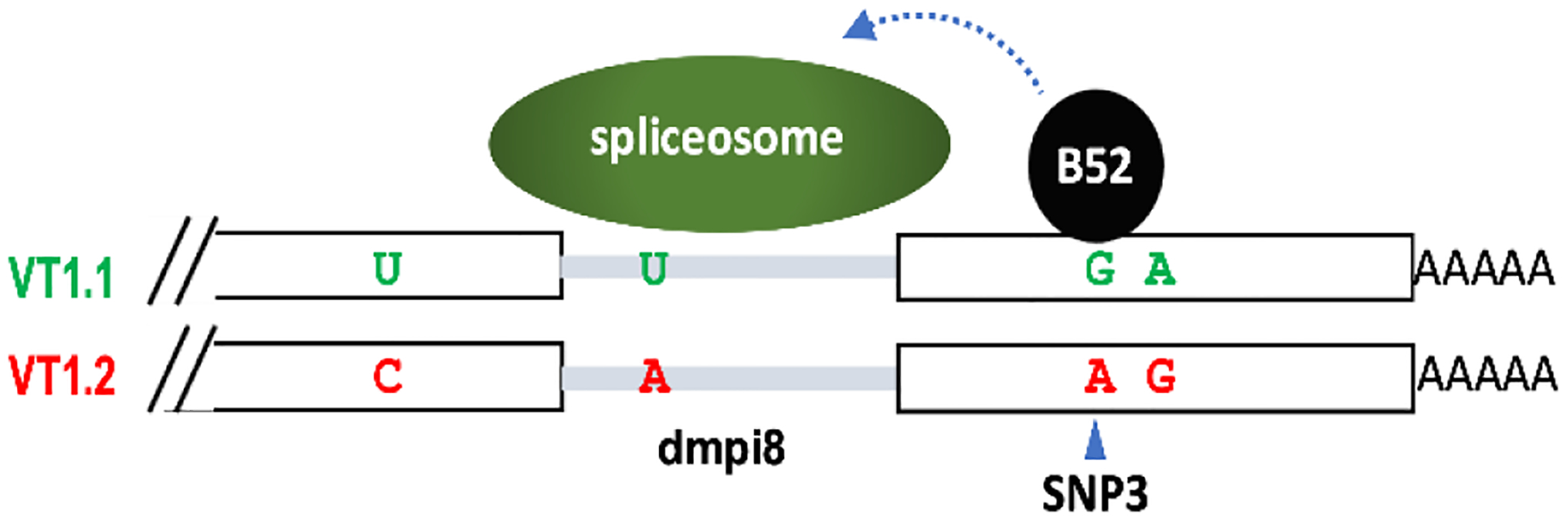
Two major *per* 3’ UTR haplotyes for natural populations of flies along the eastern coast of the United States. SNP3 is critical to dmpi8 splicing efficiency; when a G is present, it enhances binding of the B52 splicing factor, which stimulates overall dmpi8 splicing.

As an initial test case we evaluated the sleep patterns of natural populations of *D. melanogaster* from the eastern coast of the United States, spanning from Vermont to Florida^91^. Differences in the daily distribution of activity as a function of latitude were not observed. However, two major haplotypes of the *per* 3’ UTR, termed VT1.1 and VT1.2, that include four single nucleotide polymorphisms were identified (herein termed SNP1, SNP2, SNP3 and SNP4) (Fig. 4). SNP3, which is either an A or G, had the most significant effect on dmpi8 splicing and midday siesta, whereby SNP3G has higher dmpi8 splicing efficiency and diminished midday siesta compared to SNP3A. Since all dmpi8 introns are flanked by the same suboptimal 5’ and 3’ss (Fig. 3), dmpi8 splicing remains temperature sensitive irrespective of which SNP3 is present. Thus, the main thing SNP3G does is to increase the basal and peak splicing efficiency of dmpi8 but still within a physiologically relevant thermal sensitive range.

Further work showed that SNP3 is part of a binding site for B52/SRp55^92^, a member of the highly conserved serine/arginine (SR) family of splicing factor proteins. Binding of B52/SRp55 to the *per* 3’UTR is stronger when SNP3 is a G compared to A, explaining why SNP3G enhances dmpi8 splicing efficiency and reduces midday activity. Whether other SR proteins also bind the *per* 3’ UTR and modulate dmpi8 splicing efficiency is not clear. Although a distinct mechanism from that described for B52 binding to the *per* 3’ UTR in *Drosophila*, body temperature cycles in mammals can drive rhythmic phosphorylation of SR proteins that underlie thermosensitive regulation of global alternative splicing programs^93^. Thus, temperature can regulate pre-mRNA splicing in a myriad of ways, including modulating RNA secondary structure, miRNA levels and gene expression changes in splicing regulatory factors.

### *D. melanogaster* from Africa and Australia show parallel clinal changes in dmpi8 splicing and midday siesta levels.

**Fig. 5.**
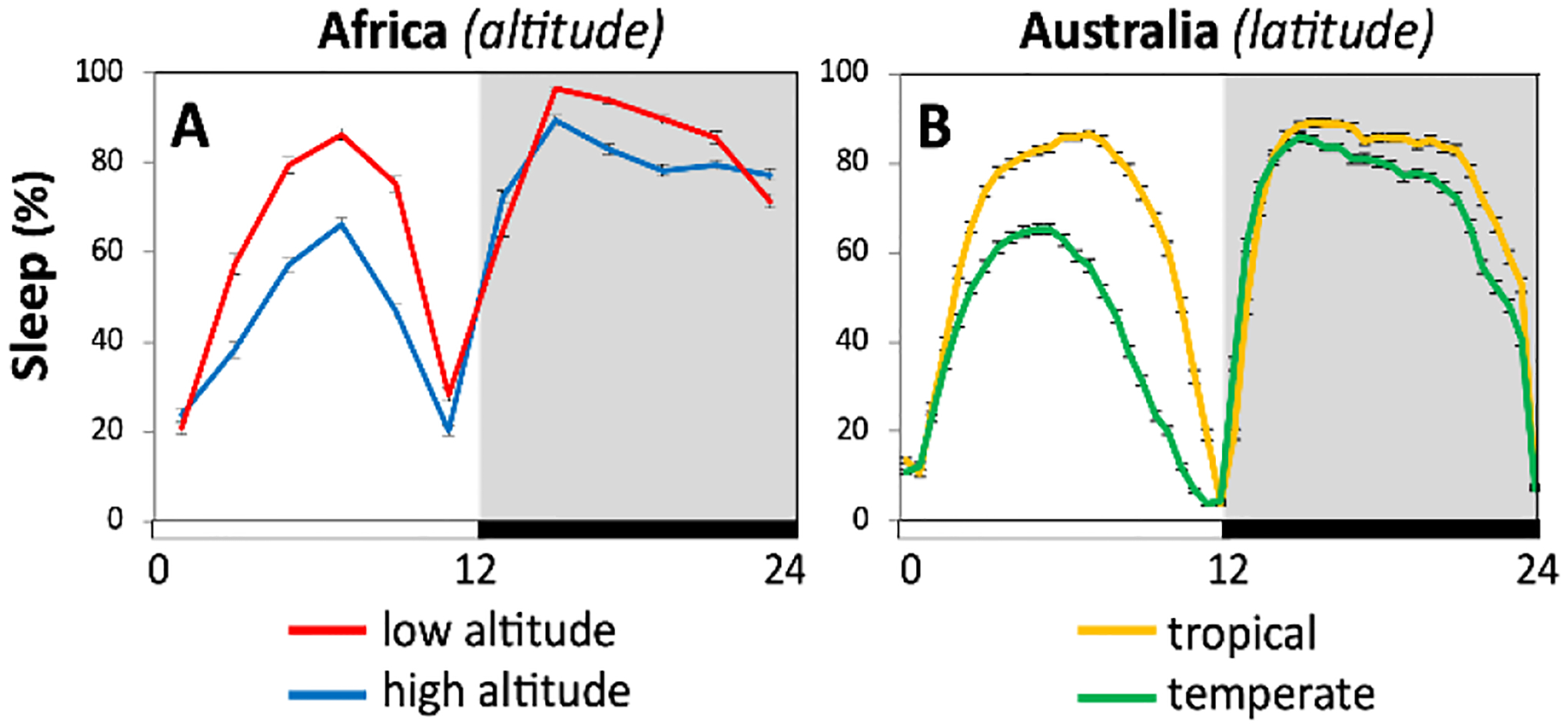
Parallel latitudinal and altitudinal clines in decreased midday siesta for natural populations of *D. melanogaster* from high altitudes in Africa (A), and temperate regions along the eastern coast of Australia (B). Shown are daily sleep profiles.

Analysis of daily wake-sleep patterns in natural fly populations from Africa and Australia revealed parallel decreases in midday siesta levels in cool adapted flies^94,95^ (Fig. 5). In the case of Africa, fly populations ranged in altitude from approximately 78 to over 3,000m above sea level. For Australia, flies were analyzed from a well-studied tropical and temperate latitudinal cline along the eastern coast^96,97^. The cool adapted flies still show increased midday siesta at warmer daily temperatures, but their overall baseline levels are lower at each temperature tested compared to the warm-adapted populations (Fig. 5). Thus, there is remarkable inter-continental congruence between altitudinal and latitudinal effects on midday siesta for flies from equatorial Africa and the eastern coast of Australia. Surprisingly however, although dmpi8 splicing efficiency figures prominently in the thermal adaptation of midday siesta as a function of altitude and latitude, the underlying mechanisms are distinct, as reviewed below.

**Fig. 6.**
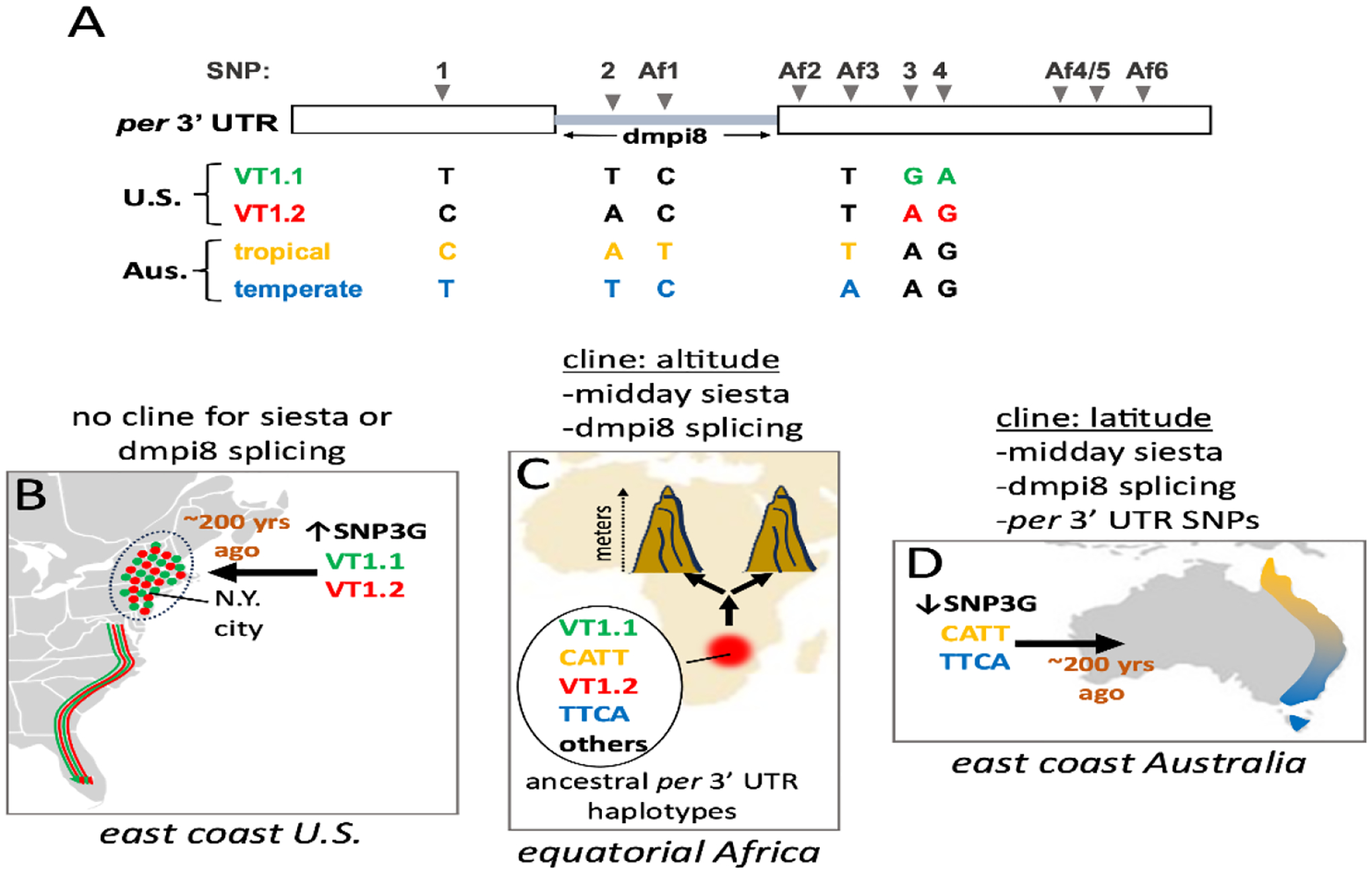
Summary of clinal relationships between midday siesta, dimpi8 splicing efficiency and *per* 3’ UTR SNPs. (A) Schematic of of the major SNPs in the *per* 3’ UTR found in natural populations of *D. melanogaster* that we sampled. (B, C, D) While all *per*3’ UTR SNPs identified to date are found in Africa, presumably also in ancestral populations (C), it is possible that the founding populations for North America (B) and Australia (D) differed in *per3*’ UTR haplotypes, perhaps explaining why latitudinal clines in dmpi8 splicing and midday siesta are observed in Australia but not the northeastern United States.

Natural populations of flies from high altitudes in Africa exhibit increased dmpi8 splicing efficiency compared to their low-altitude counterparts, consistent with their decreased midday siesta^94^. Sequencing of the *per*3’ UTR from flies representing different altitudes across eastern and western regions of equatorial Africa identified at least a dozen SNPs that generated some 10 different haplotypes of varying frequency (Fig 6A, top). This is consistent with the origins of *D. melanogaster* and the likelihood that these natural variants represent rich ancestral diversity^13^. Nonetheless, we could not find any evidence of altitudinal cline or clines in any of the *per* 3’ UTR variants either examined individually or in combination. Thus, although dmpi8 splicing efficiency was likely targeted by natural selection to adjust midday siesta as a function of altitude in equatorial Africa, this is not based on *cis*-acting elements in the *per* gene but might reside in *trans*-acting factors (e.g., Fig. 4).

A different conclusion emerged from our studies of tropical and temperate *D. melanogaster* populations representing the ‘tips’ of a well-studied latitudinal cline from the eastern coast of Australia^96,97^ (Fig. 6). Monthly averages for the tropical regions range from 21–31°C, whereas forthe temperate region it is 8–16 °C. Unlike the natural populations we examined from the eastern coast of the United States and Africa where SNP3G is present in high frequency (approx. 50–60%), this variant is rare in the Australian populations (approx. 25%)^95^. For the SNP3A containing flies from Australia we identified two major haplotypes containing four single nucleotide polymorphisms (SNPs) in the 3’ UTR of *per* (Fig. 6A, top). Two of the SNPs were similar to those identified in flies from the eastern coast of the United States and also seen in African flies (i.e., SNPs 1 and 2). Two additional SNPs seen in Australian flies were not observed in flies from the United States but were found in African flies (i.e., Af1 and Af3). Similar to SNP3, each of these four SNPs (i.e., SNP1, SNP2, Af1 and Af3) were also limited to one of two nucleotide possibilities. The two most abundant of these haplotypes exhibit a reciprocal tropical-temperate distribution in relative frequency. In tropical regions, 75% of the populations evaluated have the C/A/T/T combination of SNPs (SNP1/SNP2/Af1/Af3). Conversely, 75% of the populations from temperate regions have the T/T/C/A combination. The other less prominent SNP3A haplotypes either did not show spatial variation or were too minor to draw any solid conclusions.

Importantly, natural populations and transgenic flies with the major tropical isoform (C/A/T/T) manifest increased daytime sleep and reduced dmpi8 splicing compared to those carrying the temperate variant (T/T/C/A)^95^. Two of the SNPs (SNP2 and Af1) fall within the dmpi8 intron, whereas SNP1 and Af3 are positioned 5’ and 3’ to the intron, respectively (Fig. 6A, top). It is currently unclear why the C/A/T/T combination leads to reduced dmpi8 splicing efficiency compared to T/T/C/A. Perhaps similar to the mode-of-action at SNP3, the C/A/T/T and T/T/C/A variants differentially affect the binding efficiencies of trans-acting splicing factors such as B52. While we cannot rule out demography as a contributing factor for the inverse geographical distribution of the T/T/C/A and C/A/T/T haplotypes, gene flow between *D. melanogaster* populations has been shown to be extensive and quite symmetrical along the Australian latitudinal cline^97^. Irrespective, the maintenance of an inverse temperate-tropical distribution for the T/T/C/A and C/A/T/T haplotypes suggests active selection.

All the *per* 3’ UTR SNPs we observed in flies from the United States and Australia are present in African populations, suggesting that the different SNPs and SNP combinations in the *dper*3’ UTRs currently observed in cosmopolitan strains reflect those originating in the ancestral African populations. The C/A/T/T and T/T/C/A variants are not prominent along the eastern coast of the United States. Likewise, the major two haplotypes we noted in the United States, VT1.1 and VT1.2, are not observed in the Australian populations we studied. Although analysis of more populations is required, it is possible that the founding *D. melanogaster* strains that swept through the United States and Australia were different (Fig. 6B, D). In this regard, it is possible that early invasion of flies with high frequency of SNP3A-containing C/A/T/T and T/T/C/A strains allowed for a more subtle thermal adaptation of midday siesta via varying dmpi8 splicing efficiency. Perhaps a high prevalence of SNP3G-containing flies with much higher baseline dmpi8 splicing efficiency creates too shallow of a thermosensitive response to create a robust cline in dmpi8 splicing efficiency and hence mid-day siesta^94^. Our findings suggest that that for a large portion of *D. melanogaster* from Australia, thermal adaptation of midday siesta along the eastern coast also involved spatial selection at the level of ancestrally derived SNPs in the *dper*3’ UTR that differentially set dmpi8 splicing efficiency. Interestingly, two other studies surveying natural populations that included the America’s, Europe, East Asia but only one population from Australia did not find a connection between latitude (in the aggregate) and daytime sleep, but they both observed that nighttime sleep behavior changes as a function of latitude^98,99^. While limits in sampling size might have contributed to a lack of correlation between daytime sleep and latitude in those studies, the combined results suggest that clines in midday siesta might be limited to certain latitude-longitude combinations and/or specific regions varying in altitude. An analysis of dmpi8 splicing efficiency and *per* 3’ UTR haplotypes might provide clues as to why clines in *D. melanogaster* midday siesta are not always observed despite other traits that clearly show thermal adaptation.

In summary, there is a remarkable parallel co-evolution in *D. melanogaster* mid-day siesta as a function of altitude and latitude from two continents. In both cases, clinal changes in dmpi8 splicing efficiency are observed, indicating this molecular mechanism is a key target of natural selection. However, in flies from Australia a major mechanism is *cis*-acting SNPs that regulate daily splicing efficiency of the dmpi8 intron, which is not apparent in African flies. This suggest that even in multi-continent parallel adaptation of a behavioral trait that involves a similar molecular step (i.e., dmpi8 splicing efficiency), there is regional variety in the solutions ‘found’ by natural selection despite shared genetic variation. Additional studies are needed to show how clinal differences in midday siesta provide enhanced fitness in the wild.

### Dmpi8 splicing modulates midday siesta *in-trans* by regulating the expression of the nearby *daywake* gene.

Despite the overwhelming evidence that splicing of the dmpi8 intron in the 3’ UTR of *per* regulates midday siesta in a manner consistent with a real-world role in thermal adaptation, the possible connection of dmpi8 to *per* function was a mystery. As noted above, flies carrying the dmpi8UP version have higher dmpi8 splicing efficiency and reduced midday siesta compared to those with dmpi8WT^40^. Strangely, the reduced daytime sleep observed in dmpi8UP flies during LD cycles continues when they are exposed for several more days in constant light (LL). Without discussing too many details, prior work showed that PER protein levels are extremely low and do not accumulate in constant light conditions; in addition, behavioral and molecular circadian rhythms are abolished in constant light^100^. Thus, changes in PER protein levels are unlikely to explain how higherdmpi8 splicing efficiency connects to lower daytime sleep. Perhaps the effects of dmpi8 splicing efficiency on daytime sleep levels is independent of *per* function?

Indeed, we showed that dmpi8 splicing efficiency regulates daytime sleep *in-trans* by modulating the levels of a small slightly overlapping reverse-oriented gene that we called *daywake* (*dyw*, originally termed the ‘0.9’ gene due to its size in kb)^101^. By a mechanism that is still not understood, splicing of the dmpi8 intron somehow increases the levels of *dyw* mRNA. We suspect that spliceosome binding at the *per* dmpi8 intron is somehow co-opted to stimulate mRNA levels of the reverse transcribed *dyw*. Irrespective, numerous lines of evidence indicate that increases in the levels of *dyw* lead to decreases in daytime sleep levels by lowering arousal thresholds to sensory modalities (i.e., visible light)^101^. The ‘fortuitous’ alignment of an intron whose splicing is enhanced by cold (dmpi8) and can act *in-trans* to stimulate expression of a nearby “anti-siesta” gene *(dyw)* might have provided *D. melanogaster* with the ability to reduce midday sleep when conditions are favorable (i.e., cool), giving it a competitive advantage in adapting to temperate climates. DYW is a member of the *takeout*-family of juvenile hormone binding proteins (JHBPs) that are part of a larger lipid carrying superfamily^102^. How it functions to control daytime sleep-wake balance is not clear. Nonetheless, pertinent to this review we have not observed natural variations in *dyw* gene sequences that might suggest a role in clinal adaptation (unpublished observations). Intriguingly, the effects of dmpi8 splicing on circadian rhythms are very small^41,103^. Thus, the overriding physiological contribution of dmpi8 splicing thermosensitivity is reflected via its ability to modulate *dyw* expression, hence generating a robust mechanism for the thermal adaptation of daytime wake-sleep balance in *D. melanogaster*.

## Conclusion

It is intriguing to ponder that the serendipitous genomic alignment of a small intron with multiple weak splice sites ‘precisely’ oriented next to an anti-siesta gene allowed *D. melanogaster* to widely colonize temperate regions of the New World, setting the stage for northeasterners such as Morgan and other early pioneers to adopt it as a cheap, easily bred, naturally available species that could be exploited for genetic analysis. The rest is history, as they say.
